# Instrumentation for quantitative analysis of volatile compounds emission at elevated temperatures. Part 1: Design and implementation

**DOI:** 10.1038/s41598-020-65472-5

**Published:** 2020-05-26

**Authors:** Célia Lourenço, Sarah Bergin, Jane Hodgkinson, Daniel Francis, Stephen E. Staines, John R. Saffell, Christopher Walton, Ralph P. Tatam

**Affiliations:** 1 Centre for Engineering Photonics, Cranfield University, Cranfield, Bedfordshire MK43 0AL UK; 2 Present Address: HSE Science and Research Centre, Harpur Hill, Buxton, Derbyshire SK17 9J UK; 3 Alphasense Ltd, Sensor Technology House, 300 Avenue West, Great Notley, Essex, CM77 7AA UK; 4 Centre for Environmental and Agricultural Informatics, Cranfield University, Cranfield, Bedfordshire MK43 0AL UK

**Keywords:** Composites, Techniques and instrumentation

## Abstract

A novel suite of instrumentation for the characterisation of materials held inside an air-tight tube furnace operated up to 250 °C has been developed. Real-time detection of released gases (volatile organic compounds (VOCs), CO_2_, NO, NO_2_, SO_2_, CO and O_2_) was achieved combining commercial off-the-shelf (COTS) gas sensors and sorbent tubes for further qualitative and semi-quantitative analysis by gas chromatography-mass spectrometry coupled to thermal desorption (TD-GC-MS). The test system was designed to provide a controlled flow (1000 cm^3^ min^−1^) of hydrocarbon free air through the furnace. The furnace temperature ramp was set at a rate of 5 °C min^−1^ with 10 min dwell points at 70 °C, 150 °C, 200 °C and 250 °C to allow time for stabilisation and further headspace sampling onto sorbent tubes. Experimental design of the instrumentation is described here and an example data set upon exposure to a gas sample is presented.

## Introduction

The growing use of composite materials within aircraft has been enabled by a wide range of materials characterisation techniques to assess their performance and behaviour in the application. We report a new technique to determine the emission of volatile organic compounds (VOCs) and lighter gases from these materials as a first step in evaluating the potential for any impact on cabin air quality under both standard and elevated temperatures. A system capable of linking emissions with air quality indoors needs to be quantitative and therefore needs to be a closed system (air-tight), requires control of flow rates and reliable and accurate analysis of gaseous emissions while ramping the temperature.

Current material characterisation techniques used in fire research and air quality assessment include pyrolysis (Py) and thermogravimetric analysis (TGA) coupled with gas analyzers, such as Fourier Transformed Infrared spectroscopy (FTIR), gas chromatography-flame ionization detector (GC-FID), gas chromatography-mass spectrometry (GC-MS), or mass spectrometry (MS)^1–4^. A limitation of Py and TGA is that they cannot quantify the emission of gases. Further techniques include headspace sampling onto canisters or sorbent tubes further analysed by thermal desorption gas chromatography-mass spectrometry (TD-GC-MS) for air quality assessment indoors and outdoors^5–8^; commercial off-the-shelf (COTS) gas sensors used in real-time gas detection indoors and outdoors^9,10^. However, reports to date fall short of a fully quantitative analysis of emissions of gases and VOCs.

This paper describes a bench top instrumentation developed for the characterisation of thermal decomposition of materials at elevated temperatures in real-time and it also serves as a feasibility test of the applicability of COTS gas sensors employed in safety monitoring or in early fire detection. This closed system with good thermal control combines two complementary techniques, COTS gas sensors and in parallel, headspace sampling onto sorbent tubes for further semi-quantitative and qualitative analysis by TD-GC-MS. The instrumentation measured the sensor output voltage and correspondent gas concentration while exposing the sample to heat inside the work tube. Independent temperature measurement of the sample was also acquired; gas temperature and humidity was measured and recorded, as well as laboratory temperature. Only one sample was analysed in this study as a demonstration of the instrument. A more complete measurement campaign is reported in Part 2 of this paper.

To the authors’ knowledge, this is the first reported system to use semi-quantitative TD-GC-MS in headspace analysis of volatiles from composites and the first to combine real-time monitoring of COTS with this analytical technique. Real-time monitoring was accomplished during the heating process, allowing the progress and associated changes in materials behaviour of the experiment to be monitored. This potentially enables additional analytical samples to be collected onto TD tubes for further detailed analysis off-line.

### System overview

Commercially available sensing technologies for indoor air quality monitoring include electrochemical sensors, metal oxide semiconductors, pellistor detectors, optical absorption and photoionisation gas sensors^11–15^. The most common sensing materials are metal oxide semiconductors, where the working principle relies on the resistance change response to the target gas. COTS gas sensors are low-cost devices and measurements can be made in real-time. However, is not always possible to reliably quantify volatiles due to cross-response issues and lack of selectivity, thus other analytical techniques – such as TD-GC-MS – are therefore used to aid on compound identification and quantification.

This hyphenated technique (TD-GC-MS) uses a mass spectrometer (MS) as the GC detector; if care is taken to control gas flows and appropriate calibration standards are used it can also be quantitative^16^. In contrast to the traditional GC-MS headspace system, a major advantage of TD-GC-MS is its increased sensitivity due to sample pre-concentration via thermal desorption. The existence of a focusing trap allows larger volumes of headspace vapour to be collected without compromising the time resolution of the GC separation stage^16^.

**Figure 1:**
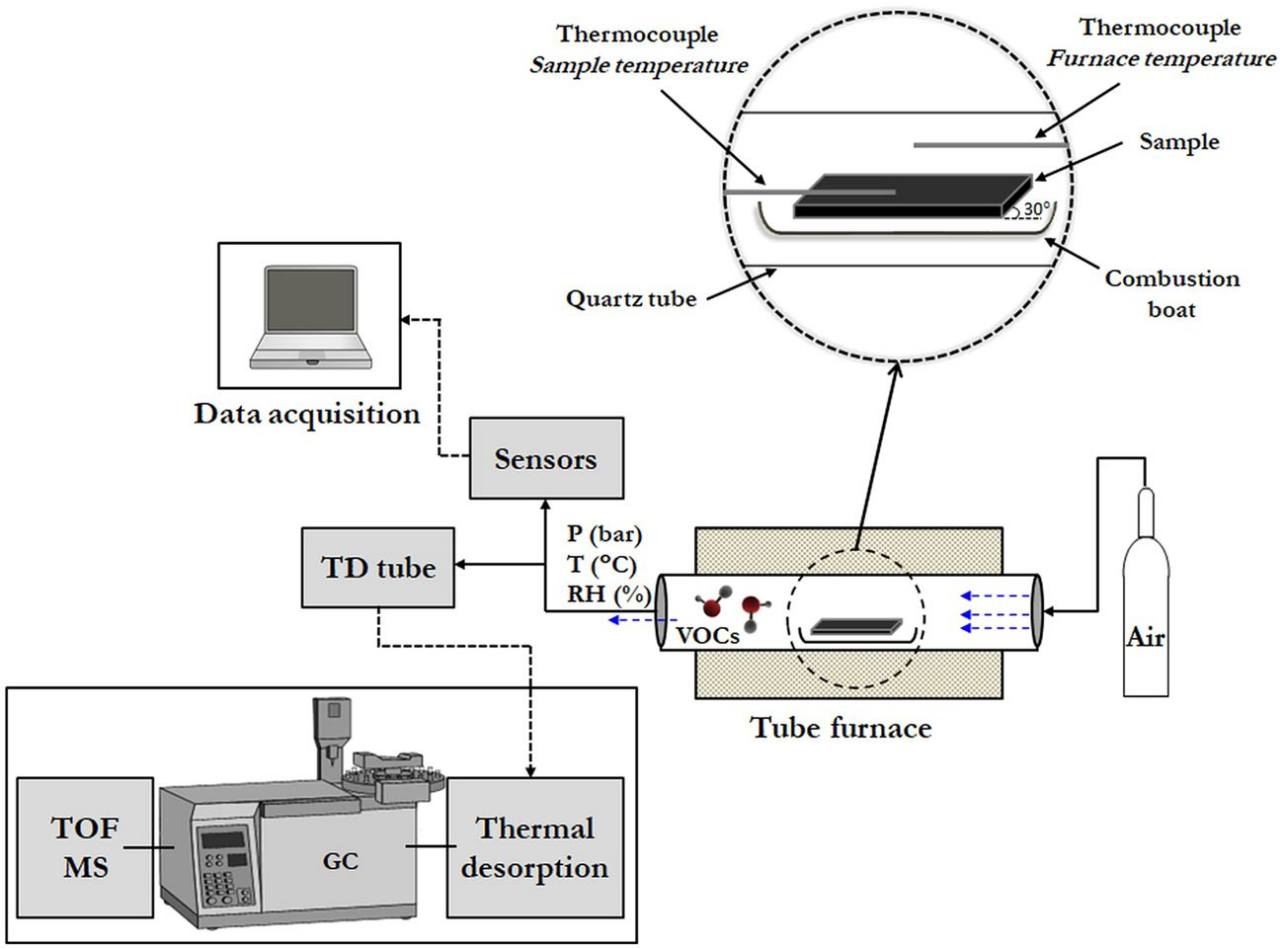
Schematic representation of the closed system for characterisation of gaseous emissions including pressure (P), temperature (T) and relative humidity (RH) control. Simultaneously gas sensing using COTS gas sensors and headspace sampling onto sorbent tubes further analysed using thermal desorption GC-MS (detection performed with a time-of-flight (TOF) mass analyser). Hydrocarbon free air: BOC products (200 bar cylinder), total hydrocarbon content (THC) < 0.1 ppm; NO_x_ < 0.1 ppm; H_2_O < 2 ppm; CO_2_ < 1 ppm.

The closed system (Fig. 1) used in the characterisation of gaseous emissions was designed to provide simultaneously gas sensing using COTS gas sensors and headspace sampling onto sorbent tubes at particular time points. The system included pressure (P), temperature (T) and relative humidity (RH) control at downstream locations within the system and this was used to monitor the gases downstream from the tube furnace and before passing through the sensors.

COTS gas sensors implemented in this study included photoionisation detection (PID) gas sensor for volatiles; non-dispersive infrared (NDIR) sensor for carbon dioxide (CO_2_); electrochemical sensors for nitric oxide (NO), nitrogen dioxide (NO_2_)_,_ sulfur dioxide (SO_2_), carbon monoxide (CO) and oxygen (O_2_). The PID gas sensor includes a short-wavelength UV lamp (Krypton lamp) that photoionises trace organic compounds (volatiles) with ionisation potentials (IE) under 10.6 eV, to produce an ionisation current which is then amplified^15,17^. It has a known cross-response to a wide range of volatiles^18^. As is standard practice, the PID sensor was calibrated with isobutylene and therefore its output is referenced to this gas.

The principle of operation of NDIR relies on the Beer Lambert law that gives the level of light transmitted through an absorbing medium such as a gas^9^. Certain gases absorb light at specific wavelengths, in this case the carbon dioxide (CO_2_) absorption band is centred at 4.2 μm (infrared region). Each sensor consists of an infrared source, optical cavity, dual channel detector and internal thermistor. The sensor comprises an active channel where the gas absorption occurs and a reference channel used to compensate for changes in the emission of the source. The sensor output (voltage) is temperature compensated as the detector used is highly sensitive to temperature differences^19^.

Electrochemical (EC) sensors enclose an auxiliary electrode, counter electrode and a working electrode where the electrochemical oxidation or reduction occurs. The charged species generated yield an electrical signal proportional to the gas concentration^20^. The oxygen sensor generates a current, which is proportional to the rate of oxygen consumption, following Faraday’s Law. The current is measured through the resultant voltage drop between a cathode and an anode^21^.

The focus of this study was to investigate the performance of composite materials under standard operating conditions and non-standard conditions such as the onset of fire. In particular, the potential for release of gases and VOCs was investigated particularly at elevated temperatures. In this study, quantitative measurements of gaseous emissions were investigated under an oxidative atmosphere while ramping the temperature at 5 °C min^−1^, with 10 min dwell points at different temperatures to allow extraction of TD samples. The sampling temperatures were chosen based on the thermal stability of the material to be analysed. A TD sample was taken at room temperature (21 °C); followed by an intermediate sample at 70 °C; a TD sample obtained at 150 °C (between the recommended operating temperature for the material of 121 °C and the glass transition temperature (Tg) at 180 °C); lastly two TD samples (200 °C and 250 °C) obtained beyond the recommended operating temperature and Tg.

## Results and discussion

### System commissioning

The sensor calibration was checked again following installation in the sensor enclosure and data acquisition/linearisation procedures had been applied. After appropriate calibration of the sensors, hydrocarbon free air was used to (a) flush the sensors and for setting up the baseline signals prior to the test and at the end of the test, (b) flush the test system and carry over any emitted exhaust gases while the tube furnace cooled down naturally to ambient temperature.

The commissioning of the whole system including measurement of the furnace outlet gas temperature and potential condensation issues; the need to use insulation plugs; response times and flow rates have been addressed.

In quartz glass tubes the heat transfer is affected by both conduction and radiation, and the temperature is lowest at the edges of the tube. The temperature distribution depends on both work tube length and inner diameter, with a temperature controlled zone in the centre of the tube^22^.

To commission the system the temperature of the controlled zone was ramped up to 250 °C. The outlet gas temperature was monitored throughout the test, increasing from 19.3 °C (at room temperature) to 20.1 °C (at furnace temperature of 250 °C). The outer wall of the quartz tube (at the exit of the furnace) was measured at 30.4 °C at a furnace operating temperature of 250 °C. At the respective operating temperatures the heat exchange onto the gas stream was negligible, and there is therefore minimal opportunity for downstream condensation. Based upon the manufacturer’s recommendation, the selected heating rate (5 °C min^−1^) was preferred since a quicker temperature rise may compromise the integrity of the quartz tube and result in damage (cracking).

Quartz tubes are known to withstand high thermal gradients, thus the manufacturer recommended use of insulation plugs made of porous alumina silicate material at high temperatures above 500 °C. These protect the rubber end seals from being over-heated and, to some degree, minimise the thermal gradient along the rest of the work tube.

In this investigation, experiments indicated that volatile compounds were highly adsorbed on the porous alumina silicate material, and these were difficult to desorb even at high temperatures (>500 °C). Therefore, to avoid contamination of the gas sample, the insulation plugs were not used throughout the tests.

**Figure 2:**
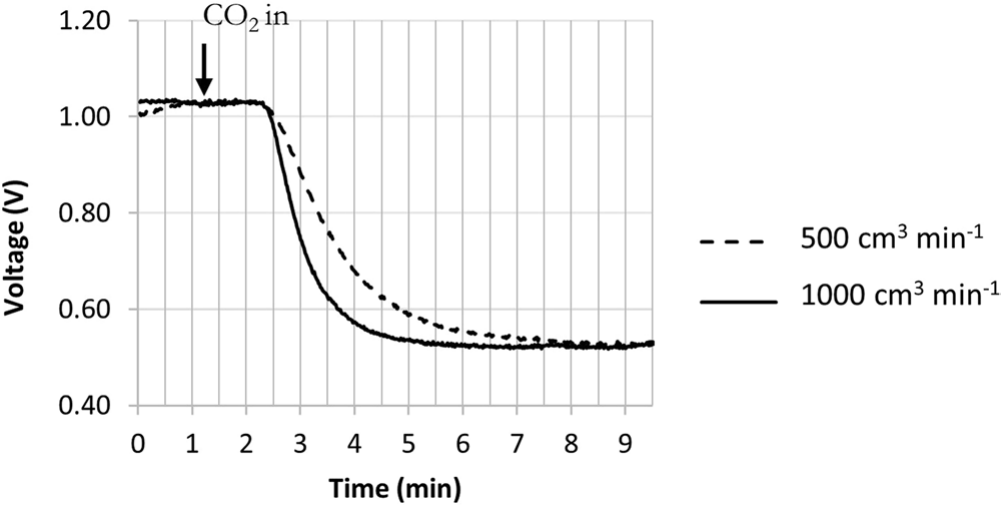
Response time of the system investigated using NDIR CO_2_ sensor at two different flow rates of standard 20% CO_2_ (200 bar gas cylinder BOC products).

Two different flow rates of standard CO_2_ have been used through the furnace to evaluate the response time of the system. Once the sensor is triggered (response time (t_90_) <40 seconds at 21 °C ambient), it takes longer to stabilise at lower flow rates (Fig. 2).

The response shows a sampling delay in the supply tubing from the CO_2_ cylinder and in the shorter tubing from the work tube to the CO_2_ sensor. This is followed by an exponential decay in measured concentration that is dominated by the time taken for the gas to mix within the work tube. For both these effects, the time taken for gases emitted from the middle of the work tube to reach the sensors is expected to be shorter than in this experiment.

### Sensor outputs

**Figure 3:**
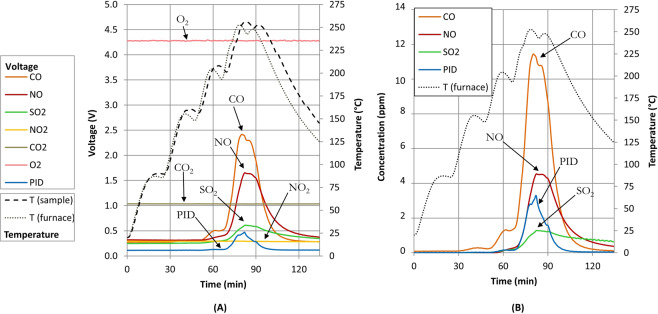
(**A**) Output voltages observed for the array of sensors CO, NO, SO_2_, NO_2_, CO_2_, O_2_ and PID (volatiles) over time. Temperature profile of the sample, *T(sample)*, and the furnace temperature *T (furnace)* measured inside the work tube. Oxygen levels remained constant over time; (**B**) Response of the sensor array (concentration expressed in parts-per-million (ppm) for CO, NO, SO_2_ and PID upon exposure to the gas sample (up to 250 °C). PID concentrations are referenced to isobutylene.

During the experiments, a flow rate of 1000 cm^3^ min^−1^ was maintained and the system allowed to stabilise for 10 min at each dwell temperature. The response of the array of sensors (Fig. 3) to the gas sample produced (while exposing the sample to heat) was recorded by measuring the change in voltage with time using the experimental system shown in the methods section.

Both the furnace temperature (i.e. the temperature inside the work tube) and the sample temperature were monitored over time, the latter being recorded by an embedded thermocouple during a separate experiment. Once the last dwell of 10 min at 250 °C had finished the furnace temperature programme was automatically turned off. Consequently, sample and furnace temperatures both decreased over time and the sensors output voltages returned to the baseline signals (Fig. 3A).

After turning off the heating, the recovery time of the system was evaluated for CO, PID, NO and SO_2_ at 39 min, 31 min, 55 min and 55 min respectively. No significant change in response was observed for the CO_2_ sensor. Oxygen levels inside the work tube remained constant over time, i.e. O_2_ output was observed at 20.6% O_2_/4.28 V ± 0.03 V.

The chronological order of the sensors triggering (Table 1) has shown that the CO sensor was the first to record a change at a furnace temperature of 124 °C (corresponds to a sample temperature of 116 °C), followed by SO_2_, PID, NO and NO_2_ at 151 °C, 186 °C, 198 °C and 237 °C respectively. The findings showed here are real, and not a result of the order in which sensors were placed in the manifold, since the CO sensor was last in the sensor chain.

**Table 1:** Response of the sensors when first triggered (baseline voltage ± 0.005 V) and respective measured temperatures.

Sensor	T (°C) furnace	T (°C) sample	Voltage (V)
CO	124	116	0.282
SO_2_	151	147	0.253
PID	186	173	0.114
NO	198	185	0.331
NO_2_	237	238	0.295

The sample temperature shown in Fig. 3A was measured in a separate experiment and shown to have a peak temperature within 2 °C of the peak furnace temperature at each of the dwell points, the latter being measured by a thermocouple situated approximately 2 cm above the sample, in the air flow. A short (5 min) delay between the peak furnace temperature and the peak sample temperature at these points may be indicative of the thermal capacity of the sample and combustion boat.

The response of the sensor array upon exposure to the gas sample is illustrated in Fig. 3B. At 250 °C, our findings suggested a peak gas concentration released of 11 ppm CO, 4 ppm NO, 2.8 ppm PID (volatiles) and 1.3 ppm SO_2_ respectively.

### Thermal desorption GC-MS

Usually, VOC concentrations are too low to permit the direct measurement of an air sample, and therefore selective analyte enrichment on suitable adsorbents is necessary. The gases pass through thermal desorption tubes packed with a sorbent bed, which is known as a pre-concentration stage prior to analyse on thermal desorption GC-MS. It is common practice to use multi-bed sorbent tubes, to allow the broadest volatility range to be analysed with a single tube. In this study a dual-bed comprising 40% Tenax and 60% Carbotrap (Markes International Ltd) was used. Tenax is inert and hydrophobic (particularly useful in humid atmospheres) and has a compound adsorption range ranging from C_6/7_ to C_30_. Carbotrap picks up more volatile compounds (C_3/4_ to C_6/7_) and those that may be desorbed at higher temperatures.

A fundamental advantage of thermal desorption is that no sample preparation is required and problems associated with the use of solvents are eliminated, e.g. variable extraction efficiency and overlapping peaks.

Volatile organic compounds (VOCs) released throughout the experiments were trapped into pre-conditioned stainless-steel sorbent tubes for 5 min at a controlled flow of 100 cm^3^ min^−1^. The tubes were sealed with brass caps (fitted with one-piece PTFE ferrules) and kept at 4 °C in a refrigerator. TD sampling was performed at different time points, i.e. 21 °C, 70 °C, 150 °C, 200 °C and 250 °C and further analysed by thermal desorption GC-MS.

Prior to analysis, the tubes were spiked with 0.5 µl of internal standard, d8-toluene in methanol (100 ng μl^−1^), and then flushed with helium for 3 min. Detailed information on the gas-phase analysis determined for six samples of the same material is further described in Part 2 of this paper.

Chromatographic analyses were performed using a GC Agilent 7890 A TOF-MS system (Bench ToF –dx (DS)) equipped with a Markes ULTRA TD autosampler, and Markes UNITY thermal desorber. GC-MS data analysis was performed through the aid of AMDIS (Automated Mass Spectral Deconvolution and Identification System) software, and followed by reliable identification of compounds using the NIST (National Institute of Standards and Technology) library.

**Figure 4:**
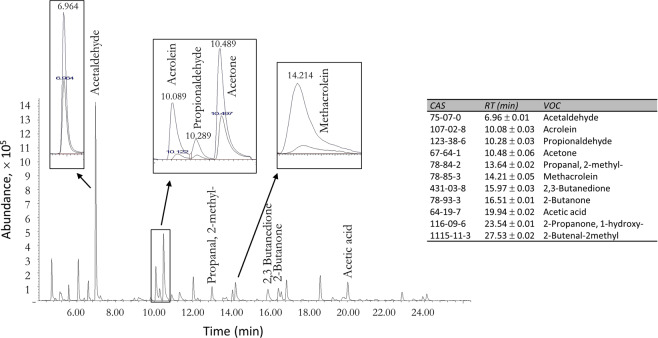
TD-GC-MS analysis: Zoomed-in chromatogram from the sample analysed at 250 °C; sections pointing out the VOC profile change with furnace temperature increase from 200 °C to 250 °C (increased abundance). Retention times (RT) (expressed in minutes) of compounds identified in this study are listed here. Compounds are ordered with respect to increasing retention time.

Volatile organic compounds (VOCs) reliably identified along with their respective chemical abstract service (CAS) registry numbers and their retention times (RT) are indicated in Fig. 4 which shows a typical chromatogram obtained from the material analysed at 250 °C and the VOC profile change with furnace temperature increase from 200 °C to 250 °C (increased abundance is observed). VOC identification with suitable signal-to-noise ratio to detect unknown VOCs was accomplished using the system described here.

## Conclusion

We have designed, built and tested an instrument to characterise gaseous emissions of materials at elevated temperatures in real-time. The tests are performed dynamically, under a constant flow of clean air, which enables the calculation of an emission rate. The developed system has the capability to deliver controlled real-time gas detection over a wide range of temperatures. To the best of our knowledge, compared to previous work this study provides semi-quantification using TD-GC-MS capabilities combined with real-time gas detection (gas sensors) in a complementary manner.

Part 1 of this paper has provided a detailed account of the methodology along with some example results from a standard aerospace composite material. Part 2 considers the results of analysis of several samples of the same material over the same range of temperatures.

We have solved and evaluated several potential issues: (1) work tube insulation plugs are problematic since we believe they promote condensation/adsorption of emitted volatiles that are then re-emitted at higher temperatures; (2) without insulation plugs, up to 250 °C (temperature in the furnace) is well-controlled for a small sample; (3) sample gas emerges at no more than 20 °C and therefore no active cooling is required; (4) we confirmed the temperature profile of the actual sample and showed that at the dwell points there was reasonable agreement between this and the temperature measured by the thermocouple installed within the furnace, which sits approx. 2 cm above the sample.

The system has some disadvantages: (1) it must be allowed to cool under air flow after testing, which can take up to 3 hours, considering 250 °C reached inside the furnace; (2) care must be taken to position and remove the sample at room temperature; (3) there is a 5 min time lag between furnace heating and sample heating at the dwell points, and 2 min time lag for the sensor response time since the gas flows inside the pipeline before reaching the sensors; (4) due to its large volume, the work tube demands a relatively high flow rate that may dilute trace species however we can make meaningful measurements well above the LOD of the sensors and within the TD-GC-MS working range; (5) due to the need to maintain the sensors at operating temperatures below 50 °C, the heating of the stainless-steel pipeline has not been employed within the system and potential minor losses of VOCs may occur.

The purpose of this system was to be a first step in enabling an assessment to be made of any potential impact on aircraft cabin air quality that might result from the emission of gases or volatiles from composite materials at standard and elevated temperatures. It should be noted that the concentrations measured using the system developed here would not be representative of the actual concentrations inside the aircraft. For practical reasons, it is necessary to measure headspace emissions that are, if anything, concentrated to render them measurable. Factors such as air flow rates and the quantity of composite material would need to be quantified, and future modelling is needed to link the emission rate in such tests with a likely concentration in the aircraft cabin.

Overall, the sensors have demonstrated a good performance to real-time detection of gases, working consistently over long periods of time (≤8 hours/day over several months). Although, in-flight environmental conditions significantly differ from ground conditions mainly in terms of pressure, thus gas sensors would have to be further tuned in order to be applied in aeronautic applications. Material characterisation, in particular air quality, plays an important role in environmental monitoring, and this system allowed the continuous and quantitative characterisation of gaseous emissions in real-time.

## Methods

**Figure 5:**
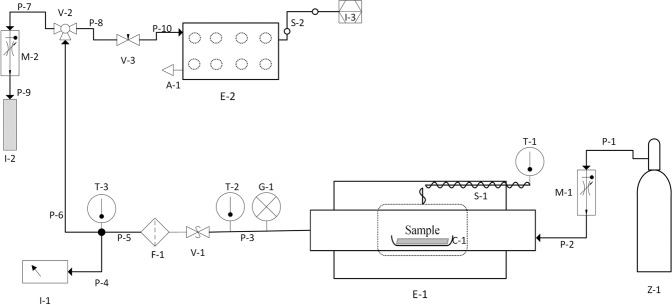
A schematic diagram of the methodology for characterisation of gas emissions at elevated temperatures in real-time using commercial gas sensors and thermal desorption tubes. Instrumentation details are listed in Table 2.

The described methodology (Figs. 5 and S1 given in Supplementary Information) allowed the characterisation of a composite material inside an air-tight horizontal tube furnace (Carbolite Gero EHA 12/300B/200) operated up to 250 °C. The real-time detection of released gases was accomplished using COTS gas sensors. The system has the capability of performing gas sampling into thermal desorption (TD) tubes implemented within the system. A detailed description of the apparatus used is listed in Table 2.

**Table 2:** Instrumentation used in the system for characterisation of gas emissions and details (n/a – non-applicable).

Instrument List	Description	Detail
A-1	Exhaust port	Swagelok (Bulkhead Union, 1/4 in)
C-1	Alumina combustion boat	Avon Green Scientific (119 × 30 × 19 mm)
E-1	Air-tight single zone tube furnace	Carbolite Gero EHA 12/300B/200
E-2	Manifold of commercial gas sensors	Alphasense
F-1	Stainless-steel in-line particulate filter	Swagelok (1/4 in, 2 μm pore size)
G-1	Pressure gauge	Swagelok (1/4 in, Female NPT)
I-1	Humidity and temperature sensor	Vaisala (HMP110 RH/T probe)
I-2	Stainless-steel thermal desorption tube	Markes International Ltd (Tenax 40%/ Carbotrap 60%)
I-3	Data acquisition	LabVIEW 2014
M-1 & M-2	Mass flow controllers	Teledyne Hastings HFC-302 (M-1 set at 1000 cm^3^ min^−1^ and M-2 at 100 cm^3^ min^−1^ accordingly)
P1–10	Electro polished stainless-steel tubing	Swagelok (1/4 in)
S-1	Heat trace	n/a
S-2	Output signals/ data	n/a
T-1	N-type thermocouple	Carbolite Gero
T-2 & T-3	EGT air probe K thermocouple	The Sensor Connection (1/8 in −27 NPT male thread)
V-1	Stainless-steel ball valve/relief valve	Swagelok (1/4 in)
V-2	Stainless-steel 3-way ball valve	Swagelok (1/4 in, HL pathway)
V-3	Stainless-steel needle valve	Swagelok (1/4 in)
Z-1	Hydrocarbon free air	BOC products (200 bar cylinder). THC < 0.1 ppm; NO_x_ < 0.1 ppm; H_2_O < 2 ppm; CO_2_ < 1 ppm.

The horizontal tube furnace (E-1 Fig. 5) consists of a quartz work tube (heated externally) with inner diameter of 55 mm, outer diameter of 60 mm and length 750 mm long. The furnace is fitted with a 3508 digital Eurotherm PID controller capable of programming multiple ramps and dwells at different times, in addition to an over-temperature control (2132 Eurotherm). The centre of the isothermal zone of the quartz tube was monitored using an N-type thermocouple (T-1 Fig. 5).

The test system was designed to provide a controlled flow (1000 cm^3^ min^−1^) of hydrocarbon free air through the furnace. The furnace temperature ramp was set at a rate of 5 °C min^−1^ with 10 min dwell points at 70 °C, 150 °C, 200 °C and 250 °C, and after completing the temperature programme the system is shut down. The temperature inside the work tube (“*furnace temperature”*, Fig. 1) was recorded at every 60 seconds using Eurotherm iTools software.

In a separate experiment, independent temperature measurement of the sample (“*sample temperature*”, Fig. 1) was monitored using a K-type thermocouple (temperature range −60 °C to +350 °C, RS Components) inserted into the sample. A temperature data logger (Pico Technology USB TC-08) recorded the average temperature every 60 seconds.

The pressure (G-1 Fig. 5) was monitored downstream of the tube furnace to ensure that the recommended maximum pressure of 0.2 bar was not exceeded. An overpressure relief valve was fitted at the exit of the furnace as a safety measure, and safety netting (Key Industrial Equipment red stretch netting 50–100 mm) fitted on both ends of the work tube to retain the end seals in case of overpressure of the system.

A stainless-steel in-line particulate filter (Swagelok 1/4 in, 2 μm pore size) (F-1 Fig. 5) was placed at the exit of the furnace to prevent any potential damage to the sensors by particles.

Temperature sensors were used at different downstream locations (T-2 & T-3 Fig. 5) to (a) ensure that the sample gases were not at a temperature that would cause any damage to the apparatus, as the temperature range of operation of the gas sensors is between −30 °C to 50 °C, and (b) to protect the TD tubes (maximum desorption temperature of 350 °C).

A combined humidity and temperature sensor (I-1 Fig. 5) (Vaisala HMP110 RH/T probe) with a detection range of 0 to 100% RH (relative humidity)/ −40 °C to +80 °C, and temperature sensors (T-2 & T-3) (EGT air probe K thermocouple, The Sensor Connection) with a detection range of −100 °C to 1300 °C and ±1.1 °C accuracy, were used to monitor the gases downstream the tube furnace and before passing through the sensors.

After the air passed over the sample it was able to carry any emitted gases to an array of COTS sensors and an analytical sampling point. A stainless-steel 3-way ball valve (V-2 Fig. 5) directed the gas flow towards the gas sensors and to pre-conditioned stainless-steel TD tubes (controlled gas flow of 100 cm^3^ min^−1^). Finally, the exhaust gases were safely vented into the fume cupboard that housed the apparatus.

Alumina combustion boats (C-1 Fig. 5) (119 × 30 × 19 mm, Avon Green Scientific) were baked off at 700 °C for 24 hours prior to use and cooled to ambient temperature inside a desiccator. Loading and unloading of the furnace was strictly performed at room temperature. The sample was sitting at an angle of 30° over the combustion boat. The set “boat+sample” was placed at the centre of the isothermal zone, and the end seals firmly closed. The sample weight and weight of the empty boat was recorded prior to the sample analysis (balance Ohaus GA200D ± 0.0001 g accuracy). Boats were weighted (boat + sample) at the end of the experiment.

The furnace cleaning procedure (high temperature cleaning) after each test was employed and a blank test was performed before each experiment with no sample in place, to ensure that the apparatus was free from contamination.

We confirmed that this apparatus can be operated safely and repeatably up to 250 °C for these composite materials. We completed a single further experiment to test whether the operating temperature could be increased up to 350 °C, however this resulted in a rapid pressure increase requiring an emergency shutdown. Further development is therefore needed to enable operation at increased temperatures.

### COTS gas sensors

Commercial off-the-shelf (COTS) gas sensors (Alphasense) (Table 3) included a photoionisation detection (PID) gas sensor (PID-AH2) (VOCs); non-dispersive infrared (NDIR) sensor for sensing carbon dioxide (IRC-A1 CO_2_); electrochemical sensors including nitric oxide (NO-A4), nitrogen dioxide (NO_2_-A43F), sulfur dioxide (SO_2_-A4), carbon monoxide (CO-A4); and oxygen (O_2_-A2) sensor. An oxygen sensor (O_2_-A2) was fitted inside the sensor system on a 4–20 mA analogue transmitter board, in order to control and monitor the oxygen levels (%) inside the tube furnace, ensuring controlled combustion of the sample under normal atmospheric conditions.

**Table 3:** Type of gas sensor and the reported limit of detection (LOD) or resolution and concentration range, expressed in parts-per-billion (ppb), parts-per-million (ppm) or % volume^17,26^ and accuracy of the sensor (Alphasense). Key: n/a – non-applicable.

Sensor	Type of sensor	LOD (ppb)	Concentration range (ppm)	Sensitivity (V/ppb)	Accuracy
PID	photoionisation	1	50	1.10 × 10^−4^	±3%
NO_2_	electrochemical	15	20	2.02 × 10^−4^	±0.5 ppm
SO_2_	electrochemical	15	50	2.69 × 10^−4^	±1 ppm
NO	electrochemical	80	20	2.96 × 10^−4^	±1 ppm
CO	electrochemical	20	<500	1.89 × 10^−4^	±1 ppm
Sensor	Type of sensor	**Resolution (ppm) Humidity sensitivity (%)**	**Concentration range (%vol)**	**Sensitivity (V/ppb)**	**Accuracy (± ppm)**
CO_2_	NDIR	500 ppm	0–20	n/a	n/a
O_2_	electrochemical	<0.7%	15–25	n/a	n/a

### Sensor system

**Figure 6:**
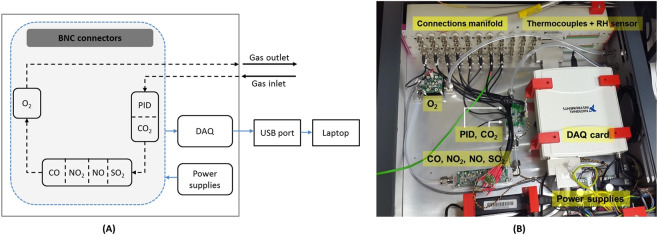
(**A**) A schematic diagram of the implemented instrumentation. Black dashed line indicates the gas flow direction through the array of sensors; (**B**) Photo of the instrument comprising an array of COTS gas sensors used in the real-time detection of gases. Key: gas sensors: PID (photoionisation detection), non-dispersive infrared (NDIR) sensor for carbon dioxide (CO_2_); electrochemical sensors for carbon monoxide (CO), nitrogen dioxide (NO_2_), nitric oxide (NO), sulfur dioxide (SO_2_); and oxygen sensor (O_2_). Data acquisition (DAQ) board; relative humidity (RH) and temperature sensor; BNC (Bayonet Neill–Concelman) connectors.

All the sensors were pre-calibrated at Alphasense, where commercially available calibration gases were used for each sensor. After appropriate calibration of the sensors, hydrocarbon free air was used to set up the baseline signals (Table S1 given in Supplementary Information). A manifold of commercial gas sensors was implemented and mounted inside a single enclosure made of aluminium (RS Components) (Fig. 6). The PID sensor was mounted on a dual board [NDIR + PID printed circuit board (PCB) (Alphasense)] together with the NDIR CO_2_ gas sensor. Four electrochemical gas sensors (NO, NO_2_, SO_2_, CO) were mounted on an analogue front end (AFE) circuit board (Alphasense) and powered to 5 V. The AFE board includes an embedded platinum resistance temperature detector (RTD), positioned adjacent to the electrochemical cells, which was used to provide temperature correction on the raw sensor responses. The internally generated temperature (*T*) dependent voltage is given by the following equation [S1] given in Supplementary Information. Where, *V* represents the measured voltage, *V*_*2*0_ _*°C*_ and *T*_*2*0_ _*°C*_ are the measured voltage at room temperature and temperature (°C) respectively^23^.

An oxygen sensor (O_2_-A2) was fitted on a 4–20 mA loop board (Alphasense). The output voltage of the current loop across a fixed resistance (249.2Ω) was measured.

A K-type thermocouple (RS Components) monitored the ambient air temperature inside the sensor enclosure. The sensors gas hoods were formed from polyvinylidene fluoride (PVDF) and included O-ring seals around the top of each sensor. All the sensors sampled gas by diffusion through a membrane into the sensor body. Fluorinated ethylene propylene (FEP) 1/4-inch tubing was used on the pipeline carrying the gas flow through the sensors and Swagelok tube fittings were used. A sampling inlet and gas outlet were fitted onto the enclosure using Swagelok fittings, in addition to a LED indicator that provides information on the operating mode of the system.

Two shielded rack mountable accessories (BNC-2090A) for 68-pin multifunction data acquisition (National Instruments Ltd) including BNC (Bayonet Neill–Concelman) connectors for analogue inputs were installed inside the enclosure. In addition, two data acquisition (DAQ) boards produced by National Instruments Ltd were used – USB-6281 M Series DAQ (16500 kS/s Analog Inputs, 24 Digital, I/O, 2 Analog Outputs) Mass Term – communicating via universal serial bus (USB) with a laptop. Two square USB 2.0 B ports have been fitted onto the sensor enclosure.

Data acquisition (output voltage, gas concentration, gas temperature, gas humidity, laboratory temperature) was automatically performed using LabVIEW 2014 software. A program has been developed in LabVIEW for this application that reads the data from the DAQs (along with labelling headers), which is appended into a text file, where one sample per second was read and one minute averages were recorded and time stamped. The front panel of the program allows the user to (1) change the name of the resulting text file (2) monitor the raw sensor outputs and environmental factors (3) monitor the determined gas concentrations in real-time (4) decide the right moment to start saving the data into a text file. The developed LabVIEW programme featured temperature correction and correction for background currents in electrochemical sensors; linearisation and temperature correction for NDIR CO_2_ gas sensor; background subtraction for PID sensor; determination of gas concentration compensated for temperature variation (if applicable).

Relative humidity and temperature probe voltage outputs (CH1 (RH%) and CH2 (T°C)) were measured and scaled signals were recorded. The 0–1 V sensor output on CH1 maps linearly to 0–100% relative humidity. The 0–1 V sensor output on CH2 maps linearly to −40 – +80 °C.

#### Determination of gas concentration

Gas concentrations were determined within the LabVIEW programme in the following manner. The voltage output was measured for the PID sensor. The resulting voltage is proportional to the gas concentration. Gas concentration determination in parts-per-million (ppm) for the PID sensor was achieved by carrying out background subtraction, followed by the division of the output voltage by the sensor sensitivity previously determined as 0.110 V/ppm.

Output voltages of working electrode (*WE*) and auxiliary electrode (*AE*) were measured for four electrochemical (*EC*) sensors (NO, NO_2_, SO_2_, CO) independently. The background voltage was subtracted from both the *WE* and *AE* measured voltage individually, given by the following equations [S2], [S3] and [S4] given in Supplementary Information. Where the value of *n* for each sensor in given in Table S1 given in Supplementary Information. The *AE* voltage was corrected for temperature changes within the range +10 to +50 °C (temperature-dependent coefficient *n*)^20^. This scaled *AE* voltage was subtracted from the *WE* voltage respectively, yielding the corrected electrochemical sensor output (*EC*_*corrected*_). In electrochemical sensors, the charged species generated yield an electrical signal proportional to the gas concentration (ppb). Thus, gas concentration determination was achieved by dividing the corrected *EC*_*corrected*_ voltage by the sensors sensitivity previously determined. The sensors sensitivity is listed in Table S1.

Voltages of active (*act*) and reference (*ref*) channels were measured for the NDIR CO_2_ sensor. The *resp*_0_ (*act*_0_*/ref*_0_) also called “*zero”* was calculated, where *act*_*0*_ and *ref*_*0*_ are the signals in the absence of the gas, i.e. the signals measured under nitrogen or hydrocarbon free air. The gas absorbance (ABS)^9,19^ is therefore determined from the sensor outputs using equation [S5] given in Supplementary Information.

The absorbance as a function of gas concentration is non-linear and the linearised gas concentration (*C*_*T*_ in % volume) was set within the LabVIEW programme and the sensor calibrated at 0–20% Vol CO_2_. The temperature corrected gas concentration, (*C*_*T*_), is determined following correction for absorbance (ABS) and *SPAN*, where *SPAN* represents the proportion of radiation that impacts on the active element of the detector and that is actually absorbed by the gas. The temperature compensated gas concentration including linearisation coefficients, *b* and *c*, is determined below (equation [S6] given in Supplementary Information). Where the values of *b* and *c* are given in Table S1. A detailed method for calculating the corrected gas concentration is given elsewhere^19^.

The oxygen sensor generates a current, which is proportional to the rate of oxygen consumption. The O_2_ sensor was calibrated in ambient air, assuming the oxygen content of air is 20.9% by volume. The sensor was calibrated following the instructions given elsewhere^24^.

Once the system had been built, the sensors were used to measure standard gases so as to check the calibration of the final system.

### Materials

The bench top system was tested with a sample (50 × 25 mm, 4 mm thickness) made of a standard carbon fibre reinforced composite material (Hexcel Hexply M21/35%/268/T700GC) (resin/resin content by weight (%)/fibre weight (gsm)/fibre type), a high strength carbon based fibre with a third generation toughened epoxy resin matrix. The M21 epoxy resin formulation is constituted of three types of epoxy resin: diGlycidyl ether bisphenol F (known as DGEBF), triglycidylether *meta*-aminophenol (known as T-GMAP), and *para*-glycidyl amine); one hardener (4,4′-diaminodiphenyl sulfone (known as DDS); and thermoplastic blends (polyether sulfone (PES) and polyamide (PA6/PA12))^2,25^.

## Supplementary information

Supplementary Information.

## Data Availability

The datasets generated during and/or analysed during the current study are available in the Cranfield Online Research Data (CORD) repository, [10.17862/cranfield.rd.9805412].
