# Supplementary material for: Instrumentation for quantitative analysis of volatile compounds emission at elevated temperatures. Part 1: Design and implementation
Source: Sci Rep. 2020 May 26;10:8700. doi: 10.1038/s41598-020-65472-5 (PMC7250926; doi:10.1038/s41598-020-65472-5)
Supplement: Supplementary file 1 — Supplementary Information. [file 41598_2020_65472_MOESM1_ESM.docx]

**Instrumentation for quantitative analysis of volatile compounds emission at elevated temperatures. Part 1: Design and implementation**

**Célia Lourenço,^1^ Sarah Bergin,^1, 2^ Jane Hodgkinson,^1*^ Daniel Francis,^1^ Stephen E. Staines,^1^ John R. Saffell,^3^ Christopher Walton,^4^ Ralph P. Tatam^1^**

^1^ Centre for Engineering Photonics, Cranfield University, Cranfield, Bedfordshire MK43 0AL, UK.

^2^ Now at: HSE Science and Research Centre, Harpur Hill, Buxton,Derbyshire SK17 9J, UK.

^3^ Alphasense Ltd, Sensor Technology House, 300 Avenue West, Great Notley, Essex CM77 7AA, UK.

^4^ Centre for Environmental and Agricultural Informatics, Cranfield University, Cranfield, Bedfordshire MK43 0AL, UK.

*Corresponding author: E-mail address: j.hodgkinson@cranfield.ac.uk ORCID ID: https://orcid.org/0000-0001-8091-7676

**Supplementary Information**

**
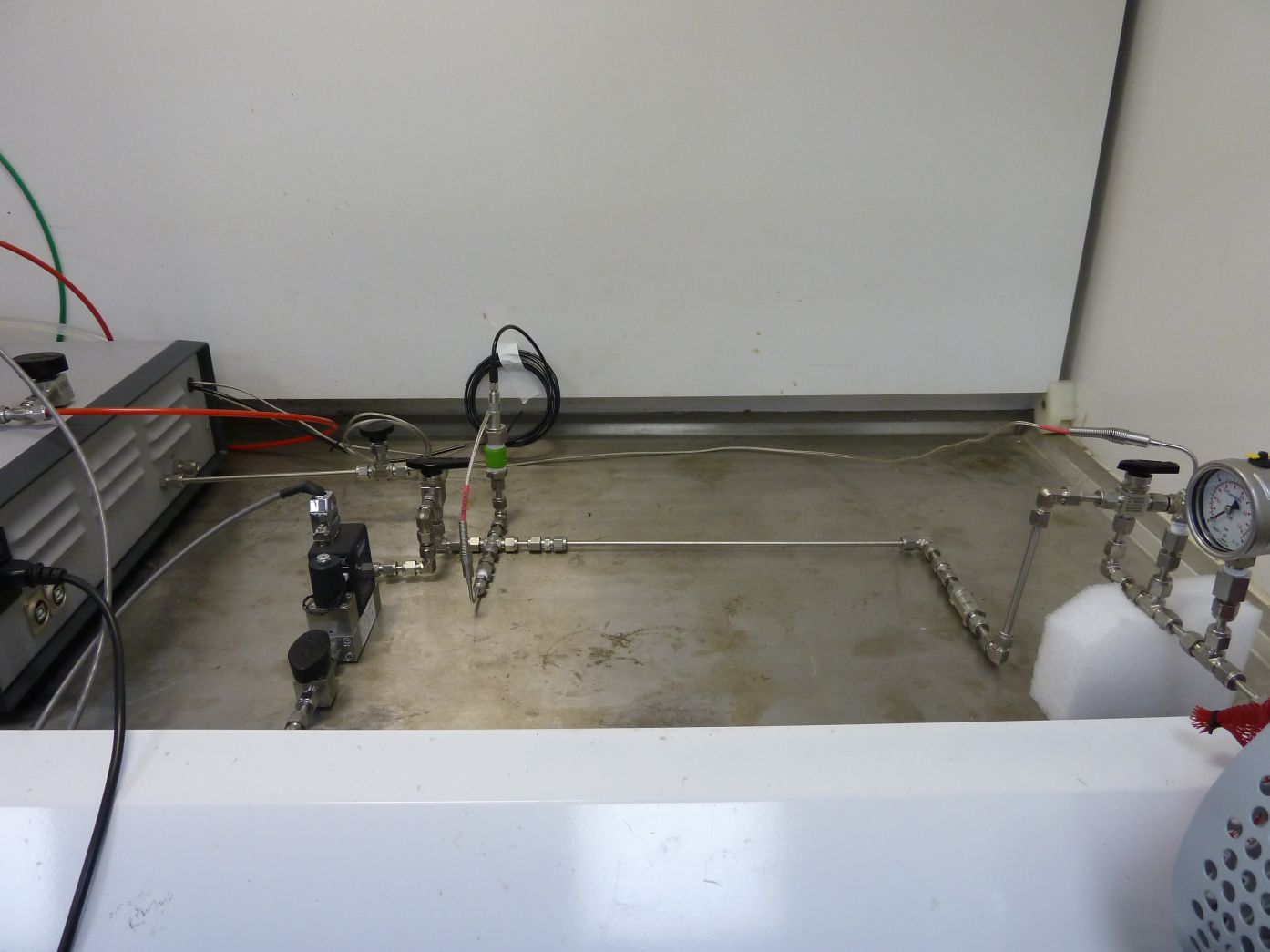
**

**Figure S1.** Photo of the bench top instrumentation developed for the gaseous analysis in real-time.

**Table S1.** Response of the sensors under hydrocarbon free air and at ambient pressure and temperature (21 °C), and the respective baseline signals (output voltages) experimentally observed. Determined sensor sensitivities expressed in V/ppb. Sensor dependent linearisation and temperature compensation coefficients are shown where relevant.

| ***Sensor*** | ***Baseline***  ***Voltage (V)*** | ***Sensitivity***  ***(V/ppb)*** | ***Linearisation coefficients*** | | | ***Temperature Compensation coefficients*** | | | | | | |
| --- | --- | --- | --- | --- | --- | --- | --- | --- | --- | --- | --- | --- |
|  |  |  |  |  |  | *Coefficients* | | *n* | | | | |
|  |  |  | ***Zero*** | ***b*** | ***c*** | ***β_0_*** | ***β_A_*** | ***10°C*** | ***20°C*** | ***30°C*** | ***40°C*** | ***50°C*** |
| PID | 0.109 | 1.10 × 10^‑4^ | n/a | | | | | n/a | | | | |
| NDIR act | 1.037 | n/a | 1.39 | 0.491 | 0.613 | 0.0024 | 0.0020 | n/a | | | | |
| NDIR ref | 0.746 |  |  |  |  |  |  | n/a | | | | |
| NO_2_ WE | 0.280 | 2.02 × 10^‑4^ | n/a | | | | | 1.18 | 1.18 | 1.18 | 2.00 | 2.70 |
| NO_2_ AE | 0.311 |  | n/a | | | | |  |  |  |  |  |
| SO_2_ WE | 0.250 | 2.69 × 10^‑4^ | n/a | | | | | 0.85 | 1.15 | 1.45 | 1.75 | 1.95 |
| SO_2_ AE | 0.261 |  | n/a | | | | |  |  |  |  |  |
| NO WE | 0.278 | 2.96 × 10^‑4^ | n/a | | | | | 1.37 | 1.37 | 1.37 | 1.37 | 1.37 |
| NO AE | 0.291 |  | n/a | | | | |  |  |  |  |  |
| CO WE | 0.263 | 1.89 × 10^‑4^ | n/a | | | | | 0.30 | 0.03 | -0.25 | -0.48 | -0.80 |
| CO AE | 0.254 |  | n/a | | | | |  |  |  |  |  |
| O_2_ | 4.312 | n/a | n/a | n/a | n/a | n/a | n/a | n/a | n/a | n/a | n/a | n/a |

Key: *act –* NDIR active channel, *ref –* NDIR reference channel; *WE* – working electrode, *AE* – auxiliary electrode, n/a – non‑applicable.

Equations

$$\left[ \boldsymbol{S}\boldsymbol{1} \right]\boldsymbol{V=}\boldsymbol{V}_{\boldsymbol{20}\boldsymbol{C}}\boldsymbol{+0.0010\times}\left( \boldsymbol{T-}\boldsymbol{T}_{\boldsymbol{20}\boldsymbol{C}} \right)$$

${\left[ \boldsymbol{S}\boldsymbol{2} \right]\boldsymbol{WE}}_{\boldsymbol{corr}}\boldsymbol{=}\boldsymbol{WE}_{\boldsymbol{measured}}\boldsymbol{-}\boldsymbol{WE}_{\boldsymbol{background}}$

$${\left[ \boldsymbol{S}\boldsymbol{3} \right]\boldsymbol{AE}}_{\boldsymbol{corr}}\boldsymbol{=}\boldsymbol{AE}_{\boldsymbol{measured}}\boldsymbol{-}\boldsymbol{AE}_{\boldsymbol{background}}$$

$${\left[ \boldsymbol{S}\boldsymbol{4} \right]\boldsymbol{EC}}_{\boldsymbol{corrected}}\boldsymbol{=}\boldsymbol{WE}_{\boldsymbol{corr}}\boldsymbol{-}\left( \boldsymbol{AE}_{\boldsymbol{corr}}\boldsymbol{\times n} \right)$$

$$\left[ \boldsymbol{S}\boldsymbol{5} \right]\boldsymbol{ABS=1-}\left[ \frac{\boldsymbol{act}}{\boldsymbol{ref\times}\boldsymbol{resp}_{\boldsymbol{0}}} \right]$$

$${\left[ \boldsymbol{S}\boldsymbol{6} \right]\boldsymbol{C}}_{\boldsymbol{T}}\boldsymbol{=}\left( \frac{\boldsymbol{T}}{\boldsymbol{T}_{\boldsymbol{cal}}} \right)\left\{ \left[ \frac{\boldsymbol{ln}\left( \boldsymbol{1-}\frac{\boldsymbol{ABS}_{\boldsymbol{T}}}{\boldsymbol{SPAN}_{\boldsymbol{T}}} \right)}{\boldsymbol{-b}} \right]^{\frac{\boldsymbol{1}}{\boldsymbol{c}}} \right\}$$

Definition

*V* – Measured voltage (V)

*V_20°C_* – Measured voltage at room temperature (V)

*T_20°C_* – Room temperature (°C)

*T* – Temperature (°C)

*WE_measured_* – Working electrode measured voltage (V)

*WE_background_* – Working electrode background voltage (V)

*WE_corr_* – Corrected working electrode measured voltage (V)

*AE_measured_* – Auxiliary electrode measured voltage (V)

*AE_background_* – Auxiliary electrode background voltage (V)

*AE_corr_* – Corrected auxiliary electrode measured voltage (V)

*n* – Temperature-dependent coefficient

*EC_corr_* – Corrected electrochemical sensor output (V)

*ABS* – Uncorrected gas absorbance

*act* – Voltages of active channel, NDIR CO_2_ sensor (V)

*ref* – Voltages of reference channel, NDIR CO_2_ sensor (V)

*resp_0_* – Zero, defined as act_0_/ref_0_

*C_T_* – Temperature corrected gas concentration (% volume)

*b* – Linearisation coefficient

*c* – Linearisation coefficient

*SPAN* – Proportion of radiation that has the ability to be absorbed by the target gas

β_0_ – SPAN only correction coefficient

β_A_ – ABS and SPAN correction coefficient

*SPAN_T_ –* SPAN at temperature, T

*ABS_T_* – Temperature corrected absorbance at temperature, T

*T_cal_* – Calibration temperature (°C)
